# Experimental Study of a Digital Feedback Fluxgate Magnetometer Using a Fifth-Order Single-Loop 1-Bit Sigma–Delta Modulator

**DOI:** 10.3390/s26175592

**Published:** 2026-09-03

**Authors:** Shang Lv, Jindong Wang, Yiteng Zhang, Xuanming Cui

**Affiliations:** 1 State Key Laboratory of Space Weather, National Space Science Center, Chinese Academy of Sciences, Beijing 100190, China; lvshang18@mails.ucas.ac.cn (S.L.);; 2 University of Chinese Academy of Sciences, Beijing 100049, China

**Keywords:** digital feedback fluxgate magnetometer, 1-bit Sigma–Delta modulation, oversampling ratio, system non-linearity, low-frequency noise

## Abstract

Digital fluxgate magnetometers have been widely used in deep space exploration due to their low noise, high sensitivity, and high reliability. This paper presents a digital fluxgate magnetometer using a fifth-order single-loop 1-bit Sigma–Delta modulator. With a consistent system structure, measurement range, test setup, and calculation method, the characteristics of magnetic field measurement noise and non-linear error are obtained under four OSR configurations through simulation analysis and experimental testing. The test results show that within the range of ±65,000 nT, the system achieves its optimal performance with a non-linearity of 0.024%, an RMS noise of 0.106 nT, and a noise power spectral density of 5.7 pT·Hz^−1/2^ at 1 Hz. These results indicate that increasing the OSR can effectively improve the performance of this digital fluxgate magnetometer, enabling high linearity and low noise measurement in Earth’s magnetic field.

## 1. Introduction

Fluxgate magnetometers offer high resolution and long-term stability in weak field measurements and are therefore widely used in deep space exploration, space physics, geophysical surveys, and spacecraft magnetic field monitoring [1]. Compared to Hall and magnetoresistive sensors, fluxgate magnetometers offer better low-frequency resolution and stability, making them suitable for scientific magnetic field measurements and spacecraft instrumentation [2,3]. A comparison of the performance characteristics of fluxgate magnetometers with other types of magnetometers is shown in Table 1.

Compared to other types of magnetometers, fluxgate magnetometers offer much higher sensitivity and better magnetic field resolution.

A conventional fluxgate magnetometer periodically drives the magnetic core to saturation and extracts information about the external magnetic field from the even harmonics of the pick-up signal [10]. In conventional fluxgate magnetometers, synchronous demodulation, integration, and feedback compensation are typically implemented using analog circuits, which impose strict requirements on component matching accuracy, low-noise design, offset drift, and temperature stability [11]. During long-term measurements, these factors can reduce measurement accuracy and complicate parameter adjustment [12]. By changing the analog signal processing circuit to the digital domain, circuit noise can be reduced and drift can be minimized [13]. For fluxgate magnetometers, these advantages are particularly important, as feedback accuracy and low-frequency noise performance must be maintained across a wide magnetic field range [14].

One-bit Sigma–Delta modulation is a commonly used signal processing method in digital fluxgate magnetometers. Its architecture has only two output levels, which can avoid static non-linearity caused by component mismatch, and push quantization noise to the higher-frequency region through oversampling and noise shaping, thereby improving the effective resolution within the target bandwidth [15]. However, quantization errors, loop dynamics, and feedback reconstruction errors still affect the system linearity and low-frequency noise performance.

## 2. Digital Feedback Principle and Design of the 1-Bit Sigma–Delta Modulator

### 2.1. Digital Feedback Principle and System Architecture

The fluxgate sensor consists of a soft-magnetic core, an excitation winding, a pick-up winding, and a feedback winding. The excitation winding periodically drives the core into magnetic saturation, the pick-up winding extracts the second-harmonic signal related to the external magnetic field, and the feedback winding generates a compensating field so that the sensor operates close to a zero-field condition.

During operation, the weak signal from the pick-up winding is amplified and demodulated by the pre-amplifier to obtain a low-frequency signal related to the external magnetic field. This signal is then integrated to generate the feedback signal, which is converted into a feedback current via the feedback path. This feedback structure extends the measurement range, improves the input–output linearity, and reduces the impact of sensor and forward-path gain variations on the measurement results [16]. Therefore, the conversion accuracy and stability of the feedback signal directly affect the overall performance of the magnetometer.

In this study, a fifth-order single-loop 1-bit Sigma–Delta modulator was used as the core component of the digital feedback path. It reduced the complexity of the feedback loop, improved quantization noise performance within the bandwidth of interest, and enhanced the accuracy of the feedback signal [17].

A 1-bit Sigma–Delta modulator consists of a loop filter, a quantizer, and a feedback path. The difference between the input signal and the feedback signal is processed by the loop filter and then applied to the quantizer. The resulting 1-bit data stream serves as the modulator output and is also returned via the feedback path, thereby forming a closed-loop modulation structure. For linearization analysis, the 1-bit quantizer can be represented by a unity-gain block and an additive quantization-error source $E\left(z\right)$. The modulator output can be expressed as

(1) $$Y\left(z\right)=STF\left(z\right)X\left(z\right)+NTF\left(z\right)E\left(z\right)$$

where $X\left(z\right)$ and $Y\left(z\right)$ represent the modulator input and output, respectively, and $STF\left(z\right)$ and $NTF\left(z\right)$ represent the signal transfer function and noise transfer function, respectively. For a loop filter $H\left(z\right)$, these transfer functions are given by

(2) $$STF\left(z\right)=\frac{H\left(z\right)}{1+H\left(z\right)}$$

(3) $$NTF\left(z\right)=\frac{1}{1+H\left(z\right)}$$

When the loop filter has high gain in the low-frequency region, the signal transfer function approaches unity within the signal bandwidth. Meanwhile, the noise transfer function suppresses low-frequency quantization noise and pushes it to higher-frequency regions. The oversampling ratio is defined as

(4) $$OSR=\frac{f_{s}}{2f_{B}}$$

where $f_{s}$ is the modulator sampling frequency and $f_{B}$ is the signal bandwidth. For an L-order low-pass Sigma–Delta modulator, the ideal noise transfer function can be approximated as [18]

(5) $$NTF_{L}\left(z\right)\approx {\left(1-z^{-1}\right)}^{L}$$

Equation (5) shows that an L-order low-pass Sigma–Delta modulator has L zeros near z = 1, which enhances the suppression of DC and low-frequency quantization noise.

**Figure 1:**
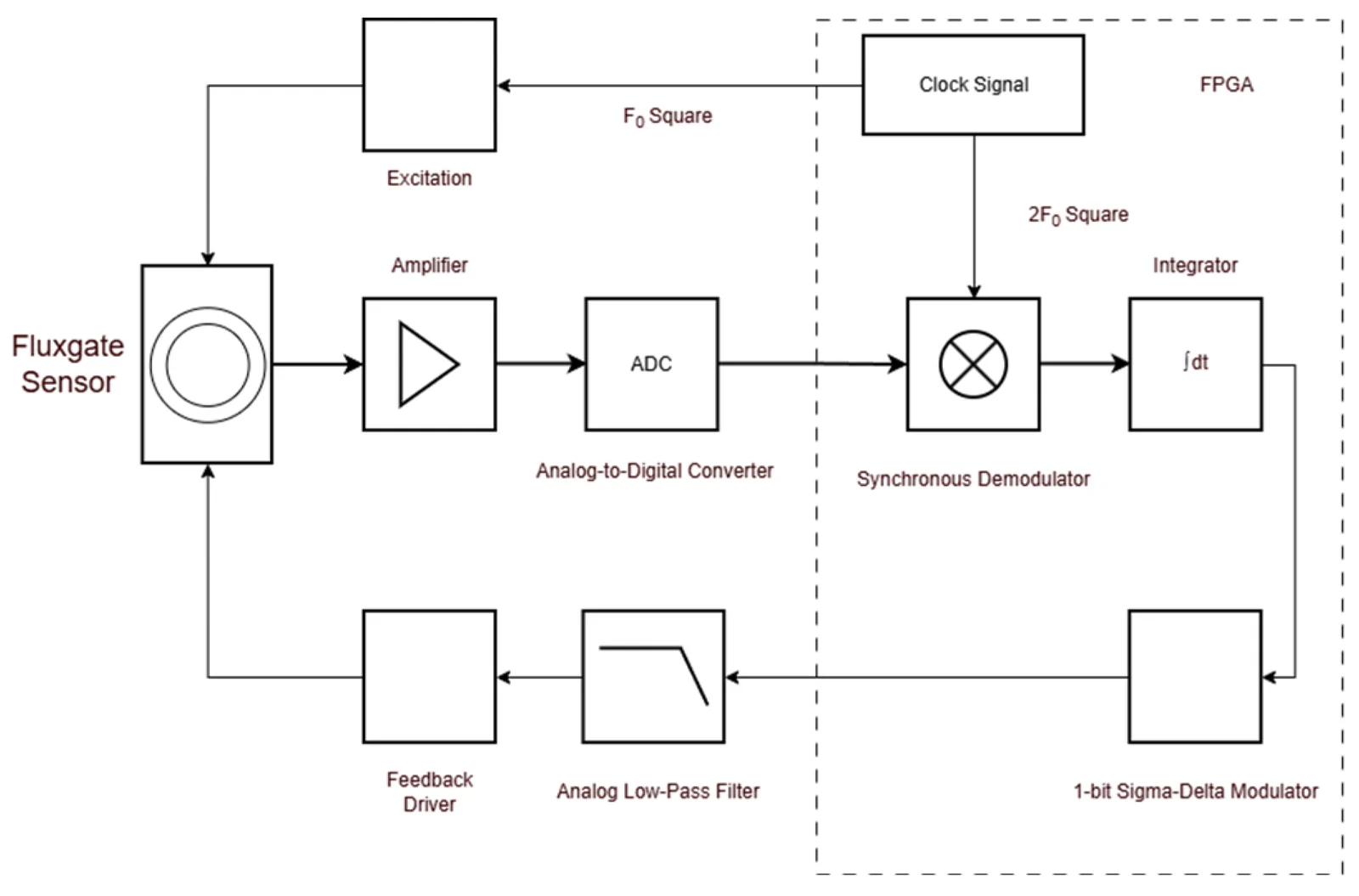
Block diagram of the 1-bit digital feedback fluxgate magnetometer.

Figure 1 shows the basic block diagram of the digital fluxgate magnetometer. The system consists of a fluxgate sensor, an excitation circuit, a pre-amplifier, an ADC, a digital processing unit, an analog low-pass filter, and a feedback driver.

### 2.2. Fifth-Order 1-Bit Sigma–Delta Modulator

**Figure 2:**
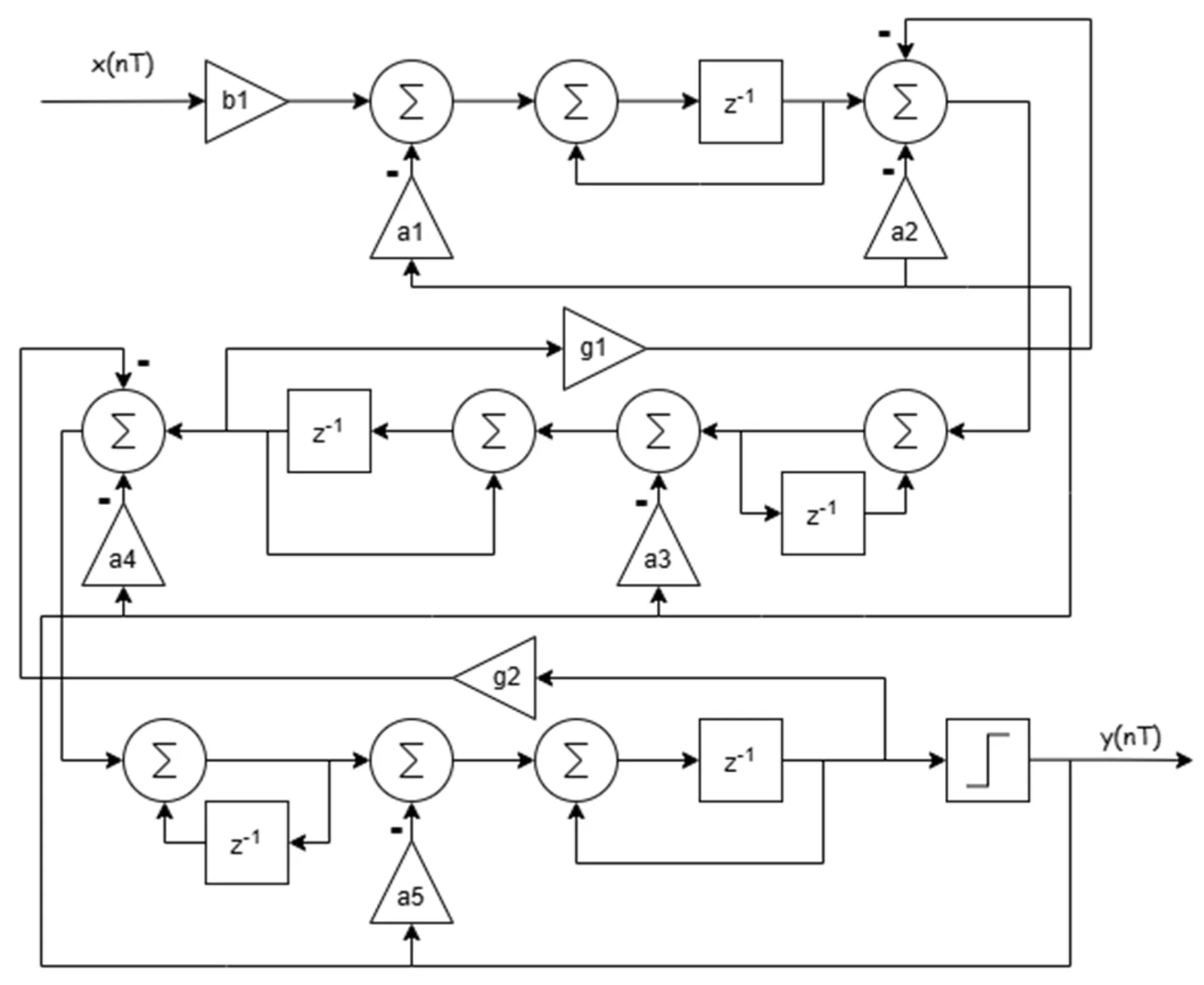
Block diagram of fifth-order single-loop 1-bit Sigma–Delta modulator.

Increasing the order of a Sigma–Delta modulator generally improves noise performance within the signal bandwidth, yet it also increases loop complexity and imposes stricter stability requirements [19]. Therefore, modulator design requires a balance between noise shaping capability, implementation complexity, and stability. In this study, a fifth-order structure was selected to achieve a compromise between low-frequency quantization noise suppression, hardware complexity, and the stable operating range. Although higher-order modulators offer superior noise shaping performance, they often increase internal signal swing, complicate circuit design, and narrow the stable input range [20]. As shown in Figure 2, the fifth-order single-loop 1-bit Sigma–Delta modulator was implemented using a cascade-of-resonators feedback (CRFB) topology.

The noise transfer function of a fifth-order low-pass modulator can be expressed as

(6) $$NTF_{5}\left(z\right)\approx {\left(1-z^{-1}\right)}^{5}$$

Equation (6) shows that the fifth-order structure has five zeros at z = 1. The noise transfer function of the fifth-order structure can be expressed as [21]

(7) $$NTF_{5}\left(z\right)=\frac{\prod_{i=1}^{5}\left(z-z_{i}\right)}{\prod_{i=1}^{5}\left(z-p_{i}\right)}$$

where $z_{i}$ and $p_{i}$ are the zeros and poles of the noise transfer function, respectively. The fifth-order noise transfer function was mapped to the CRFB topology to obtain the corresponding modulator coefficients. In this design, only $b_{1}$ was used as the input injection path, all other input injection coefficients were set to zero, and all inter-stage coefficients were set to unity.

For OSRs of 128, 512, 1024, and 2048, respectively, the corresponding fifth-order modulator coefficients were calculated, and the input amplitude and integrator states were verified to ensure that the modulator remains stable within the current input range. Since the target noise transfer function varied with OSR, a separate set of coefficients was used for each configuration. The main coefficients are listed in Table 2.

During the coefficient optimization process, the stability of the fifth-order single-loop 1-bit modulator was taken into account. The maximum gain γ of the noise transfer function was set to 1.5, satisfying [22]

(8) $$||NTF_{\infty }||=max_{\omega }\left|NTF\left(e^{j\omega }\right)\right|\leq \gamma$$

A lower gain threshold generally improves the stability of the modulator but increases the noise level. Therefore, the coefficient design requires balancing the stability margin within the signal bandwidth against quantization noise. For the four sets of coefficients, a stability verification process was employed, including noise transfer function design, pole–zero analysis, and input range checks.

**Figure 3:**
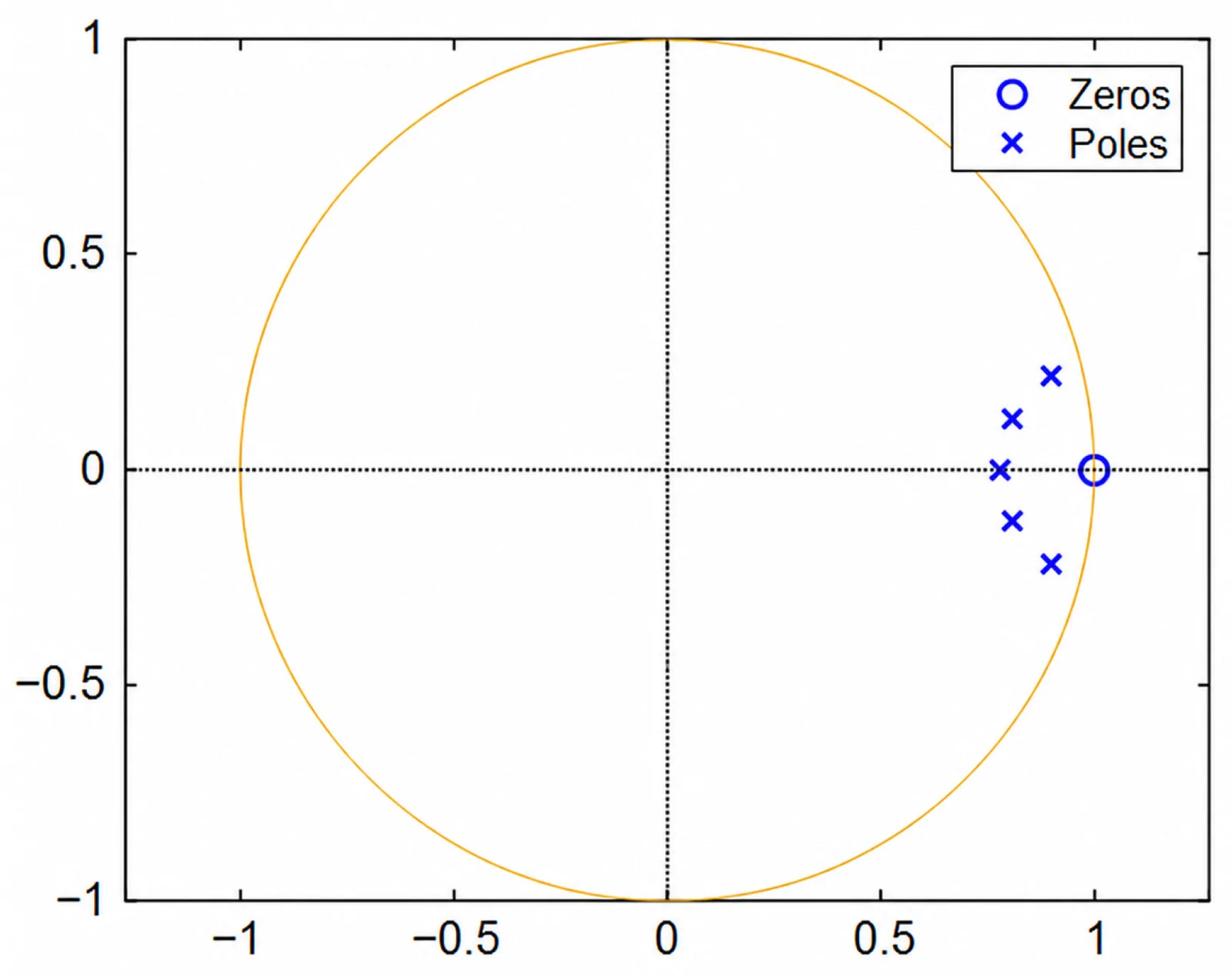
Pole–zero plot of the fifth-order single-loop 1-bit Sigma–Delta modulator at OSR = 2048.

As shown in Figure 3, when OSR = 2048, the pole–zero plot of the modulator reveals that the five zeros of the noise transfer function were located at z = 1, and all poles lie within the unit circle, indicating that the loop characteristics were stable. Similar results were obtained for the other three coefficient configurations. Therefore, in subsequent prototyping experiments, these four sets of coefficients were used to compare the system linearity and low-frequency noise.

## 3. Experimental Study of the 1-Bit Sigma–Delta Digital Feedback Fluxgate Magnetometer

In this section, a 1-bit Sigma–Delta digital feedback fluxgate magnetometer was evaluated through experimental measurements. First, a system simulation model was established to serve as a reference for parameter selection during the experimental tests. Subsequently, a fluxgate magnetometer measurement system was constructed. Under identical conditions regarding system structure, full-scale range, calibration methods, measurement setup, and operating conditions, four different OSR configurations were tested to evaluate their impact on system linearity and low-frequency noise performance.

### 3.1. System Schematic

**Figure 4:**
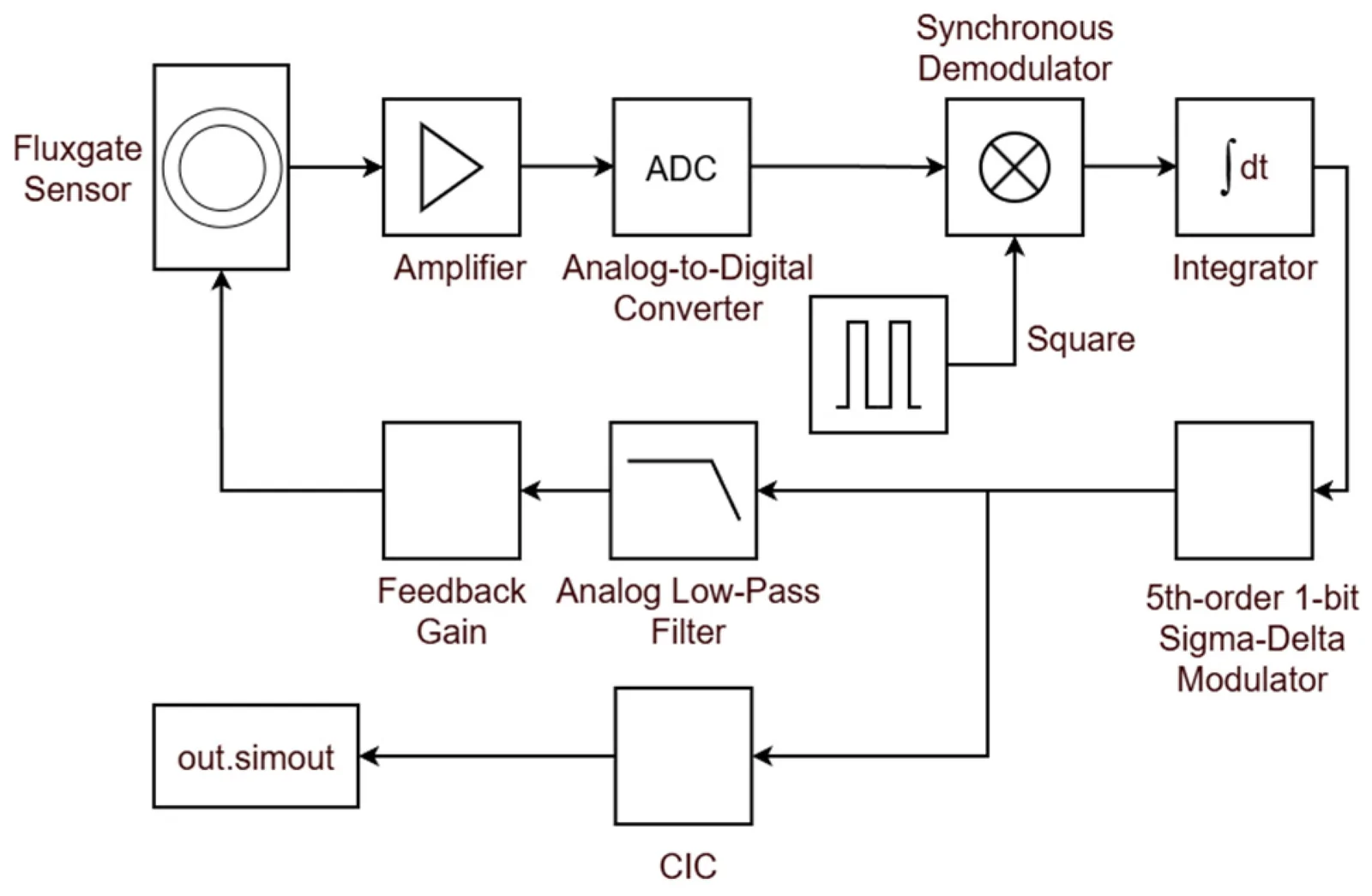
System schematic of the 1-bit digital feedback fluxgate magnetometer.

A system model of the 1-bit digital feedback fluxgate magnetometer was established, and the system performance was simulated. The system schematic is shown in Figure 4.

Using this simulation model, the influence of OSR values of 128, 512, 1024, and 2048 on the non-linearity and low-frequency noise characteristics of the 1-bit digital feedback fluxgate magnetometer system were analyzed. The system mainly consisted of a fluxgate sensor, an amplifier, an A/D converter, a synchronous demodulator, an integrator, a fifth-order single-loop 1-bit Sigma–Delta modulator, an analog low-pass filter, feedback gain, and a CIC down-sampling filter. The operating frequency of the entire system was 1.024 MHz. The fifth-order single-loop 1-bit Sigma–Delta modulator adopted the CRFB structure shown in Figure 2. During the simulation process, the modulator was configured according to the modulation coefficients listed in Table 2, and the changes in system performance under different OSR values were analyzed. The output data was down-sampled through the CIC output filter.

### 3.2. Circuit Architecture and Signal Processing Flow

**Figure 5:**
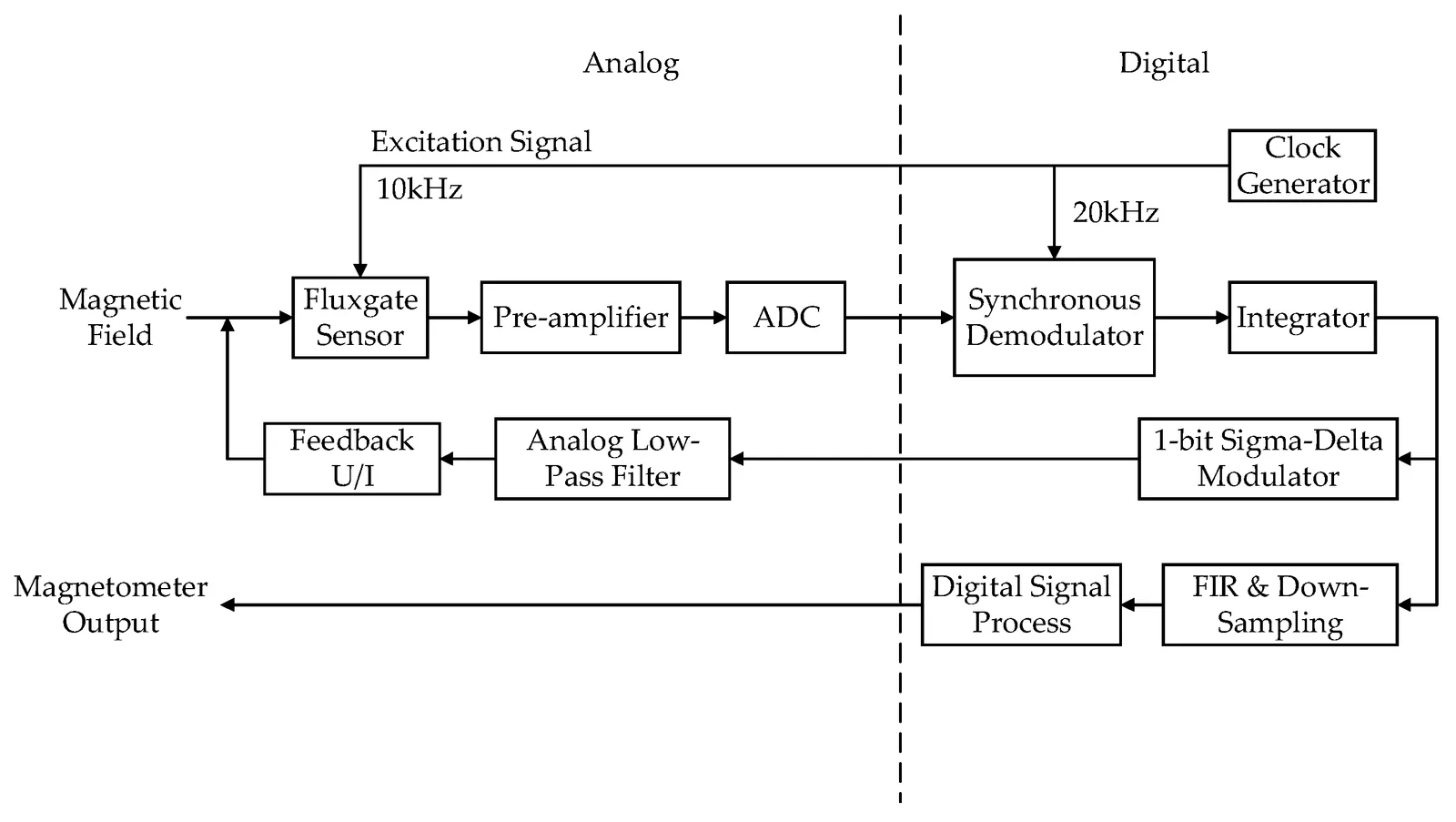
Block diagram of the magnetometer system.

Corresponding to the system simulation model, the experimental hardware system consisted of an analog circuit and an FPGA-based digital signal processing unit, as shown in Figure 5. The excitation circuit used a 10 kHz square signal to drive the fluxgate sensor. The pick-up signal acquired by the sensor was amplified by an AD620 instrumentation amplifier, digitized by an AD7982 ADC, and then transferred to the FPGA.

Within the FPGA, the sampled signal is demodulated by the synchronous demodulator using a 20 kHz reference signal to extract the second-harmonic signal. After processing by a digital integrator, this signal is split into a measurement path and a feedback path. In the measurement path, magnetic field measurement data was obtained through digital filtering and down-sampling. In the feedback path, the fifth-order single-loop 1-bit Sigma–Delta modulator with four different OSR configurations generates a 1-bit data stream. This data stream then passes through an analog low-pass filter and a feedback driver to produce a feedback current used to compensate for the external magnetic field.

**Figure 6:**
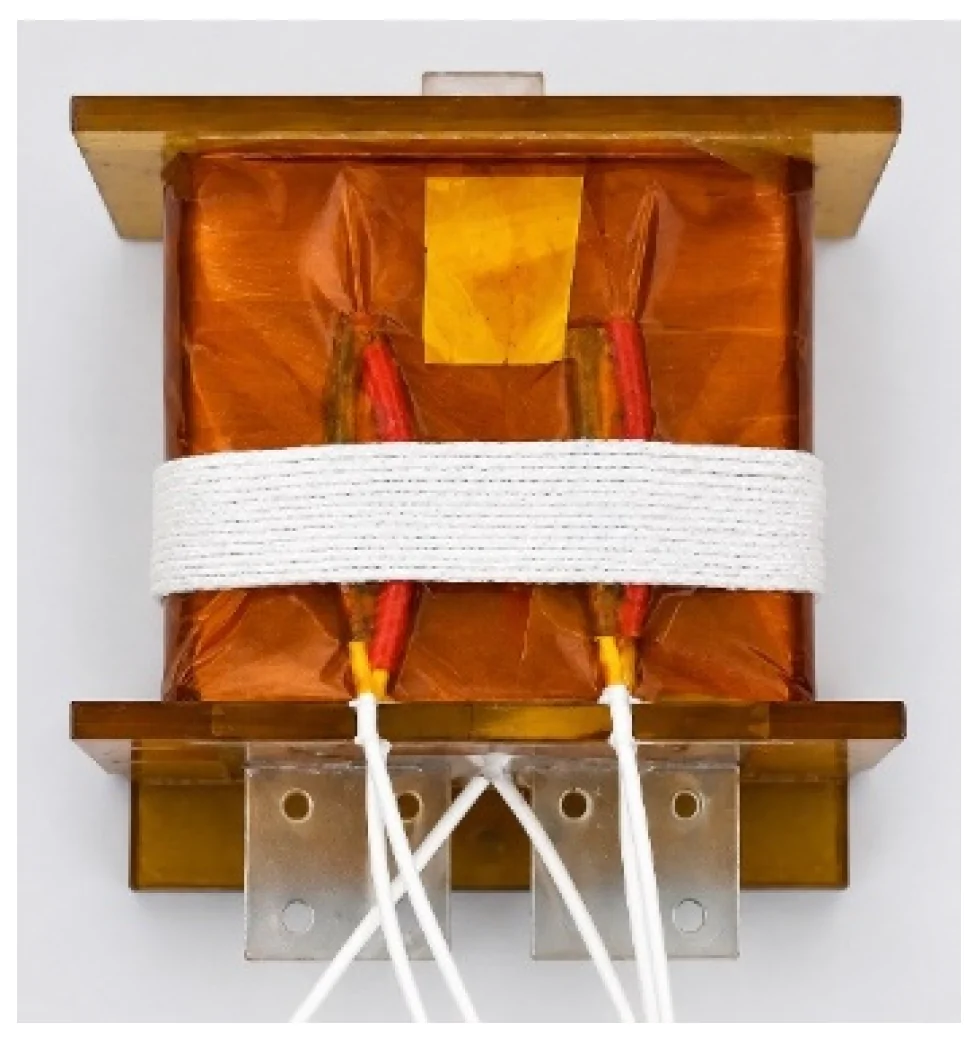
Photograph of the fluxgate sensor.

The sensor used in the experimental system is shown in Figure 6. It is a single-axis Permalloy ring-core fluxgate sensor with separated excitation, pick-up, and feedback windings.

**Figure 7:**
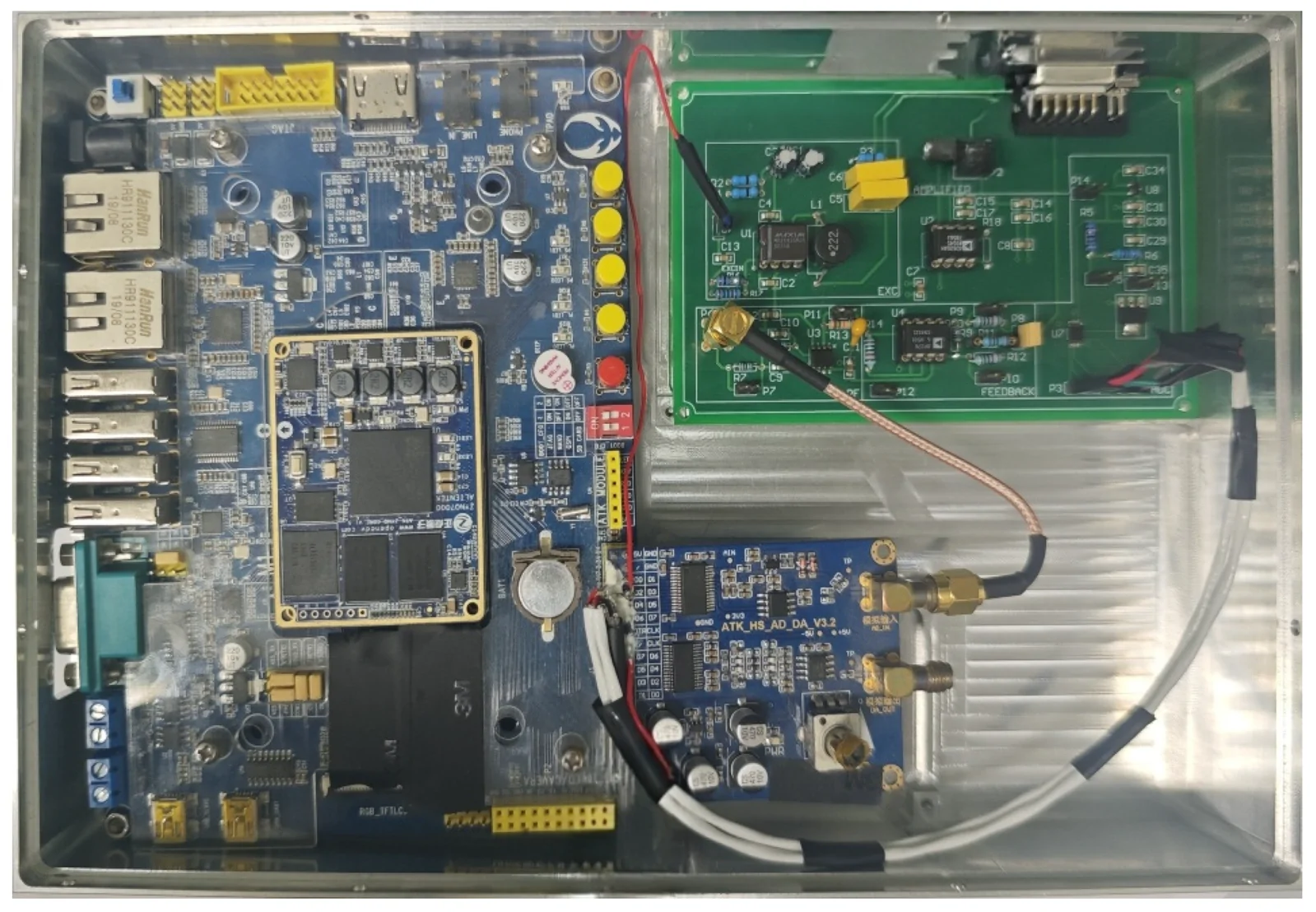
Photograph of the electronic components of a fluxgate magnetometer.

Figure 7 shows the hardware implementation of the magnetometer electronics. Digital signal processing was implemented on a Xilinx ZYNQ-7020 APSoC development board. A custom analog circuit board handled signal conditioning and feedback current drive.

### 3.3. Key Parameters

To ensure a controlled comparison of the four different OSR configurations, all experiments employed the same system architecture, input magnetic field range, excitation signal frequency, sampling ratio, performance evaluation metrics, calibration methods, and data processing procedures for testing system linearity and noise performance. Due to the hardware limitations, OSR = 2048 was the highest configuration that can be reliably achieved in the present test system. The main parameters of the experimental system are listed in Table 3.

### 3.4. Measurement Results and Analysis

#### 3.4.1. Test Setup

**Figure 8:**
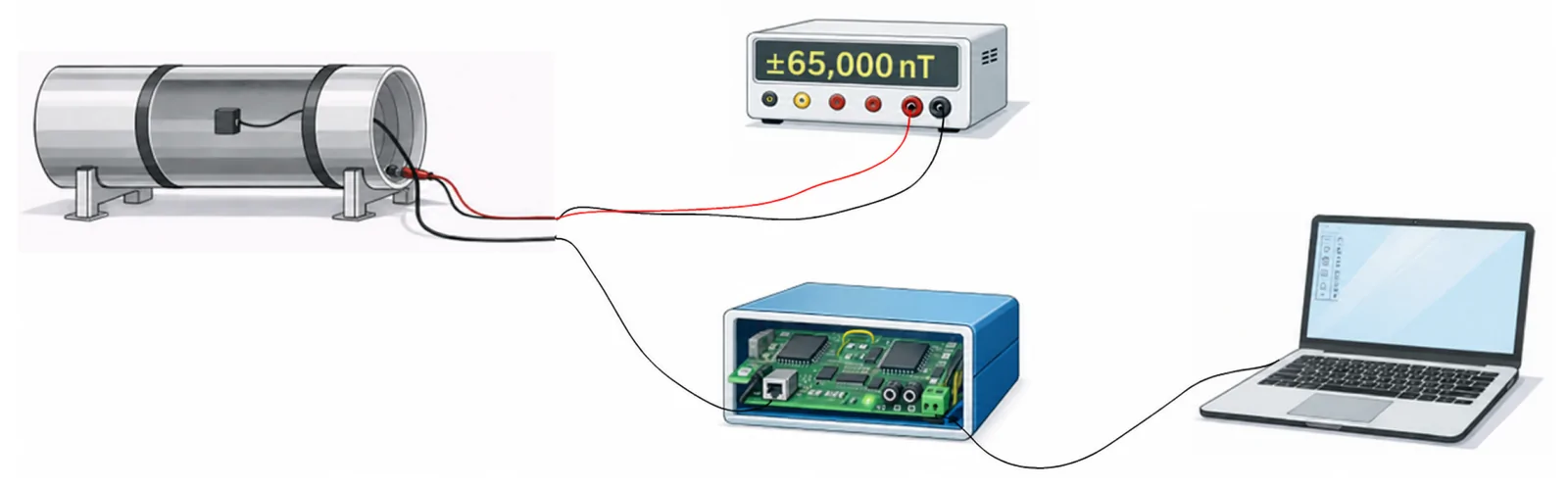
Schematic of the measurement system.

**Figure 9:**
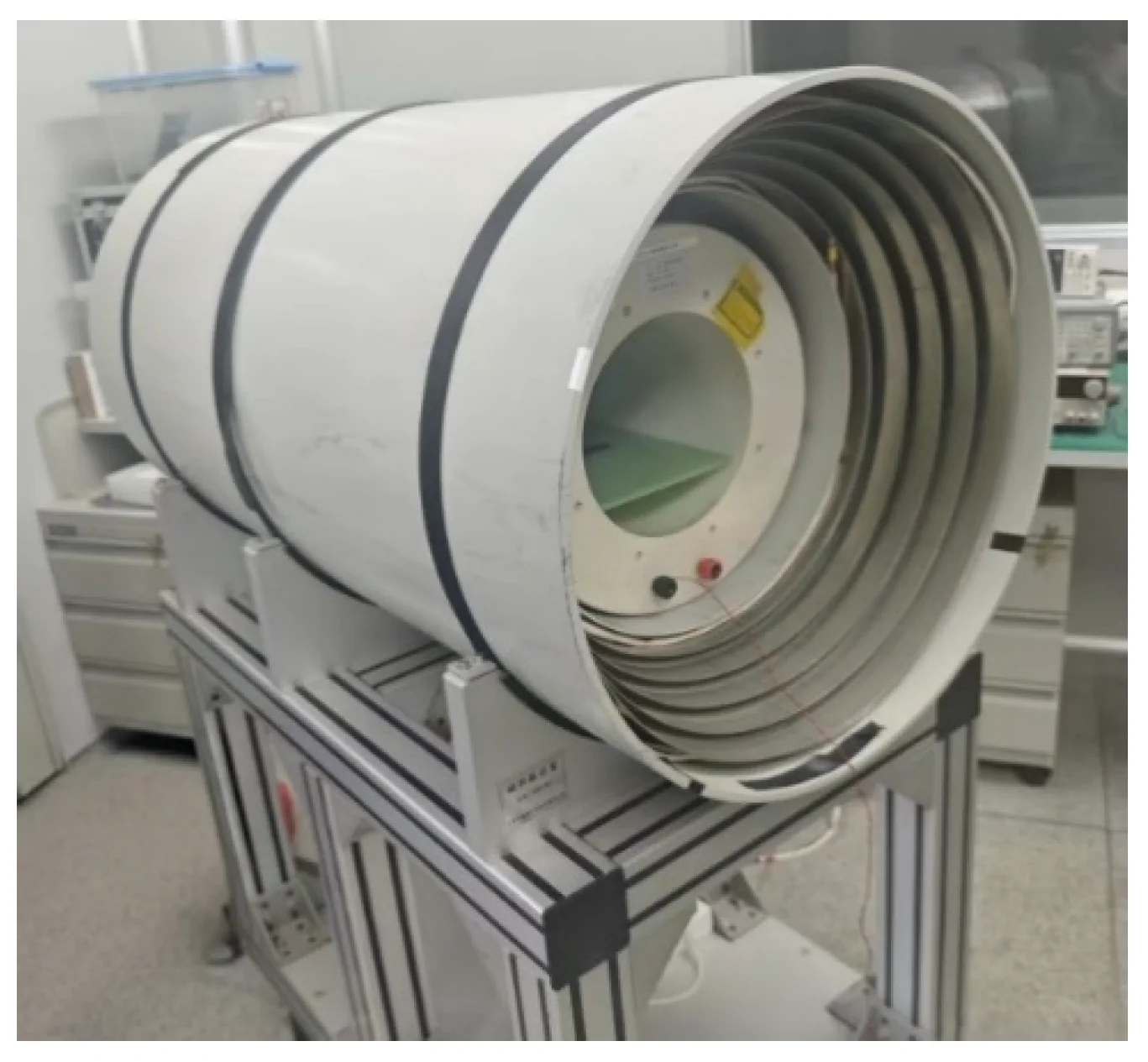
Photograph of the magnetic shielding can and Helmholtz coils.

The test setup consisted of a magnetic shielding can, a Helmholtz coil, a DC power supply, a magnetometer, and a data acquisition system. The magnetic shielding can was adopted to minimize the influence of the environmental magnetic field. The Helmholtz coil could generate controllable static magnetic fields within ±65,000 nT. The fluxgate sensor was placed within the uniform field region of the coils, with its measurement axis aligned with the direction of the applied magnetic field. With all other test conditions held constant, tests were conducted using only four different OSR configurations. Figure 8 shows the connection scheme of the measurement system. Figure 9 shows a photograph of the magnetic shielding can and Helmholtz coils used in the test setup.

#### 3.4.2. Linearity Measurement

The system non-linearity was measured using a Helmholtz coil under four different OSR configurations, with the magnetic field swept from −65,000 nT to 65,000 nT at a step size of 10,000 nT. Using a least-squares linear fitting method, the relationship between the applied magnetic field and the magnetometer output was obtained:

(9) y=k·x+b

where x is the applied magnetic field, y is the magnetometer output, and k and b are the fitted scale factor and offset, respectively. The system non-linearity was calculated based on the maximum fitting residual relative to the full-scale range.

**Figure 10:**
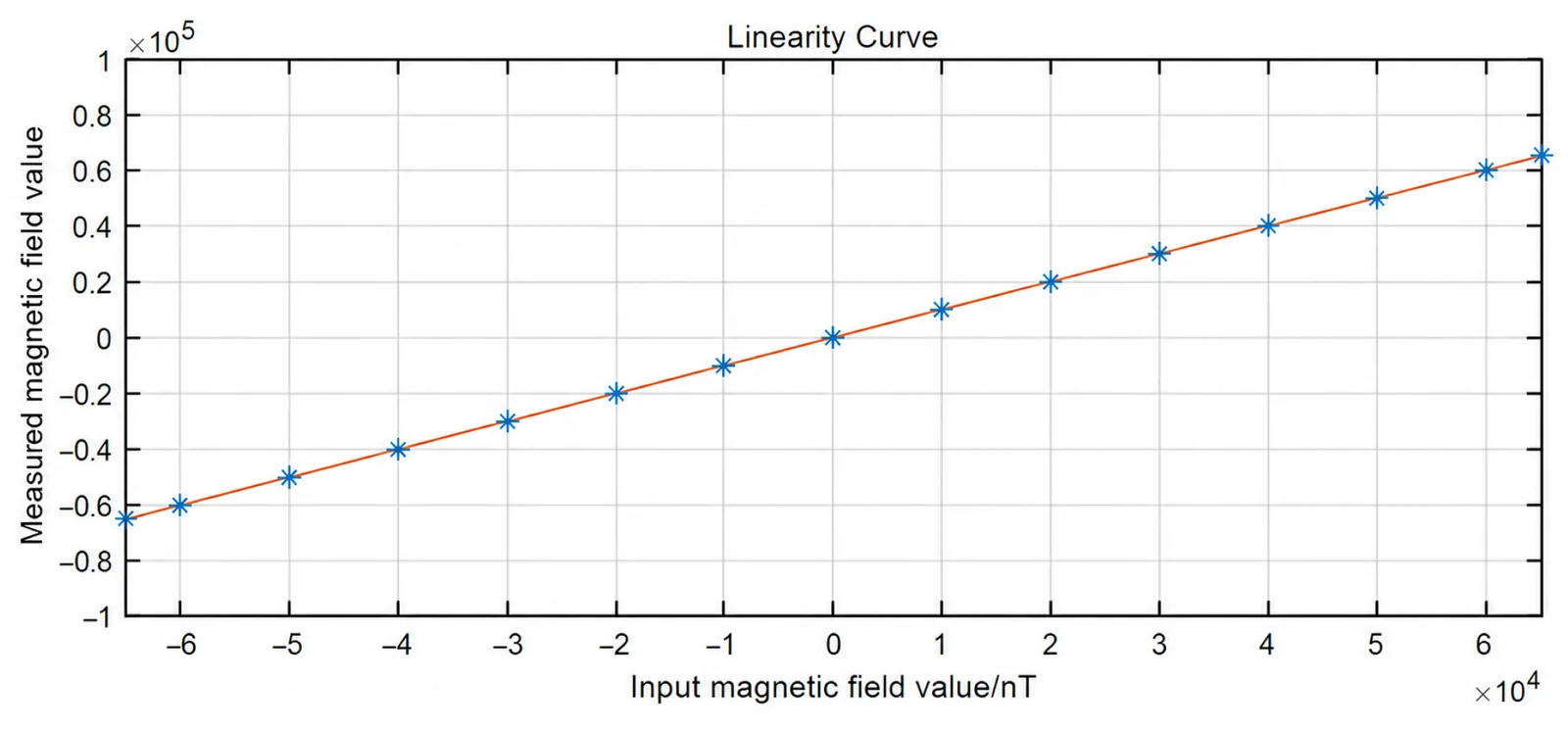
Linearity curve for OSR = 2048.

**Figure 11:**
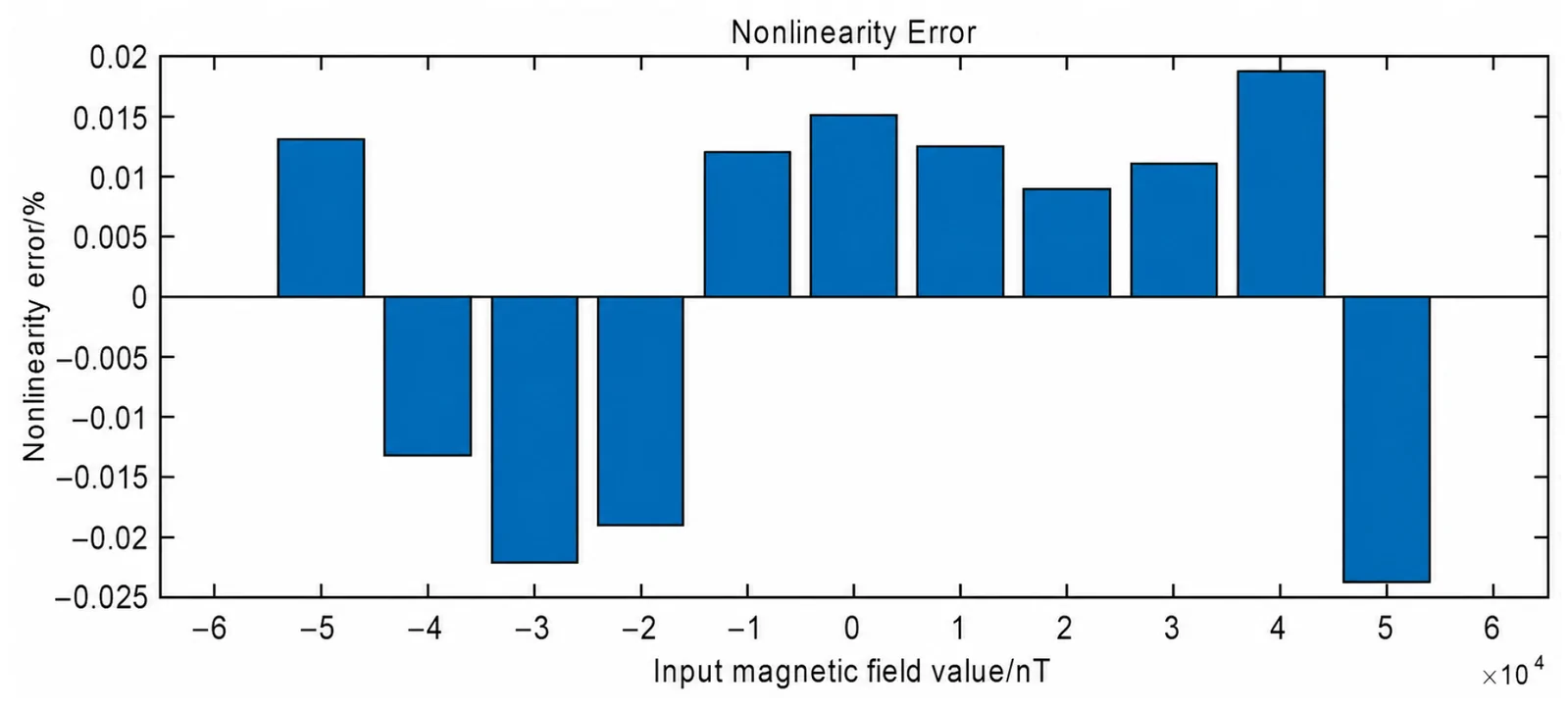
Non-linearity error for OSR = 2048.

Figure 10 and Figure 11 show the linear curve and non-linearity error obtained under the OSR = 2048 configuration, respectively. Across the entire measurement range, the measured response agrees well with the fitted straight line. The non-linearity error fluctuates near zero and does not exhibit a clear monotonic dependence on the applied magnetic field. The corresponding system non-linearity is 0.024%.

Table 4 lists the system non-linearity using four different OSR configurations. As the OSR increased from 128 to 2048, the system non-linearity decreased from 0.050% to 0.024%. Since all coefficient configurations were tested on the same hardware platform using the same calibration scheme, measurement range, and data acquisition program, the results clearly demonstrate how different OSR configurations affect system non-linearity.

#### 3.4.3. Noise Measurement

For the noise measurement, the output data was recorded for 30 s at an output data rate of 100 Hz, yielding a total of 3000 sampling points. After subtracting the mean from each data set, the noise RMS was calculated in the time domain. Fast Fourier transform was then applied to obtain the single-sided noise power spectral density. The noise power spectral density at 1 Hz was obtained by averaging 11 spectral values, including the band closest to 1 Hz and five adjacent bands on either side of that band.

**Figure 12:**
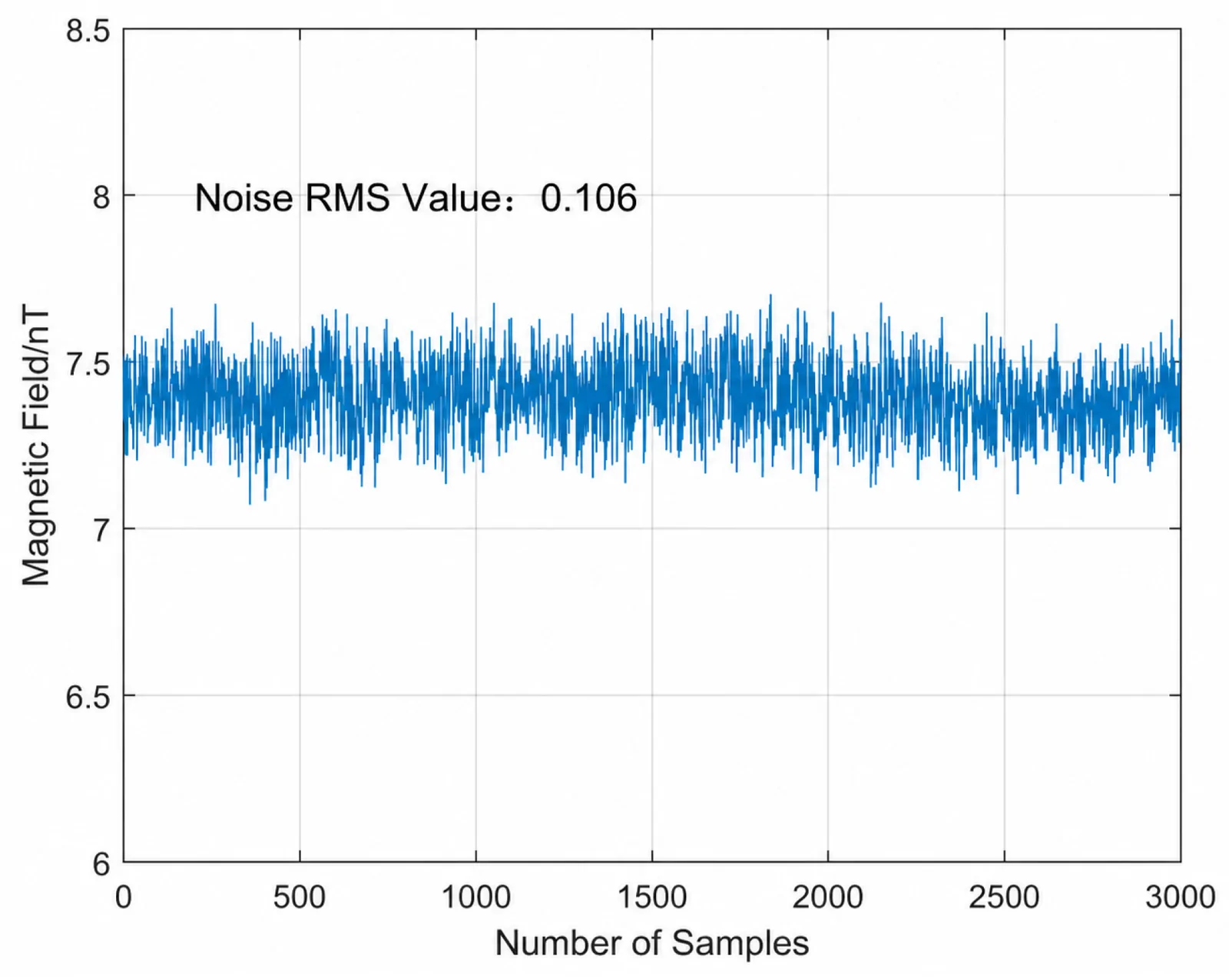
Time domain noise waveform for OSR = 2048.

Figure 12 shows the time domain noise waveform obtained with an OSR of 2048. The output values fluctuate randomly around their mean, with no apparent periodic variations or systematic drift. After subtracting the mean, the noise RMS was 0.106 nT.

**Figure 13:**
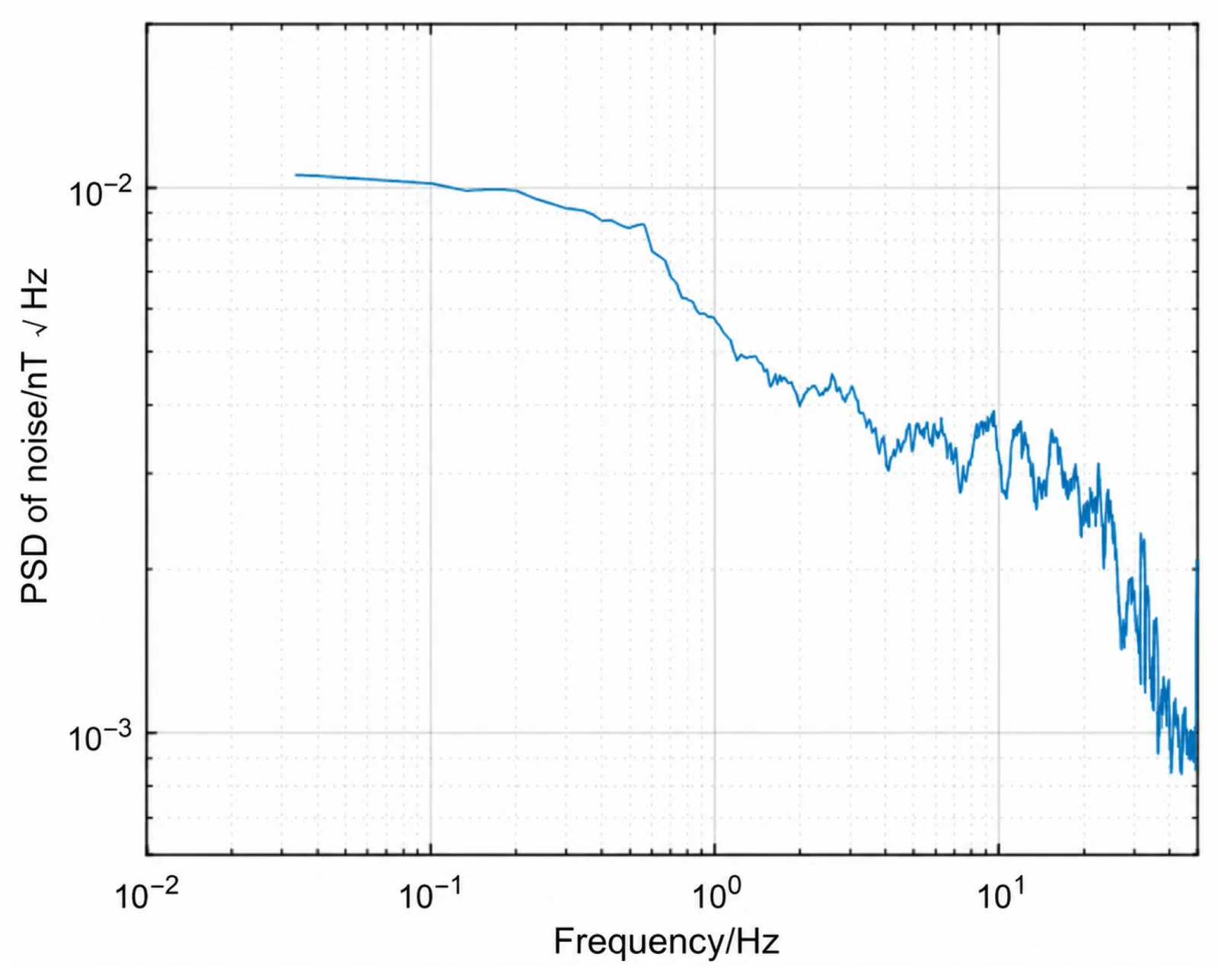
Noise power spectral density for OSR = 2048.

**Figure 14:**
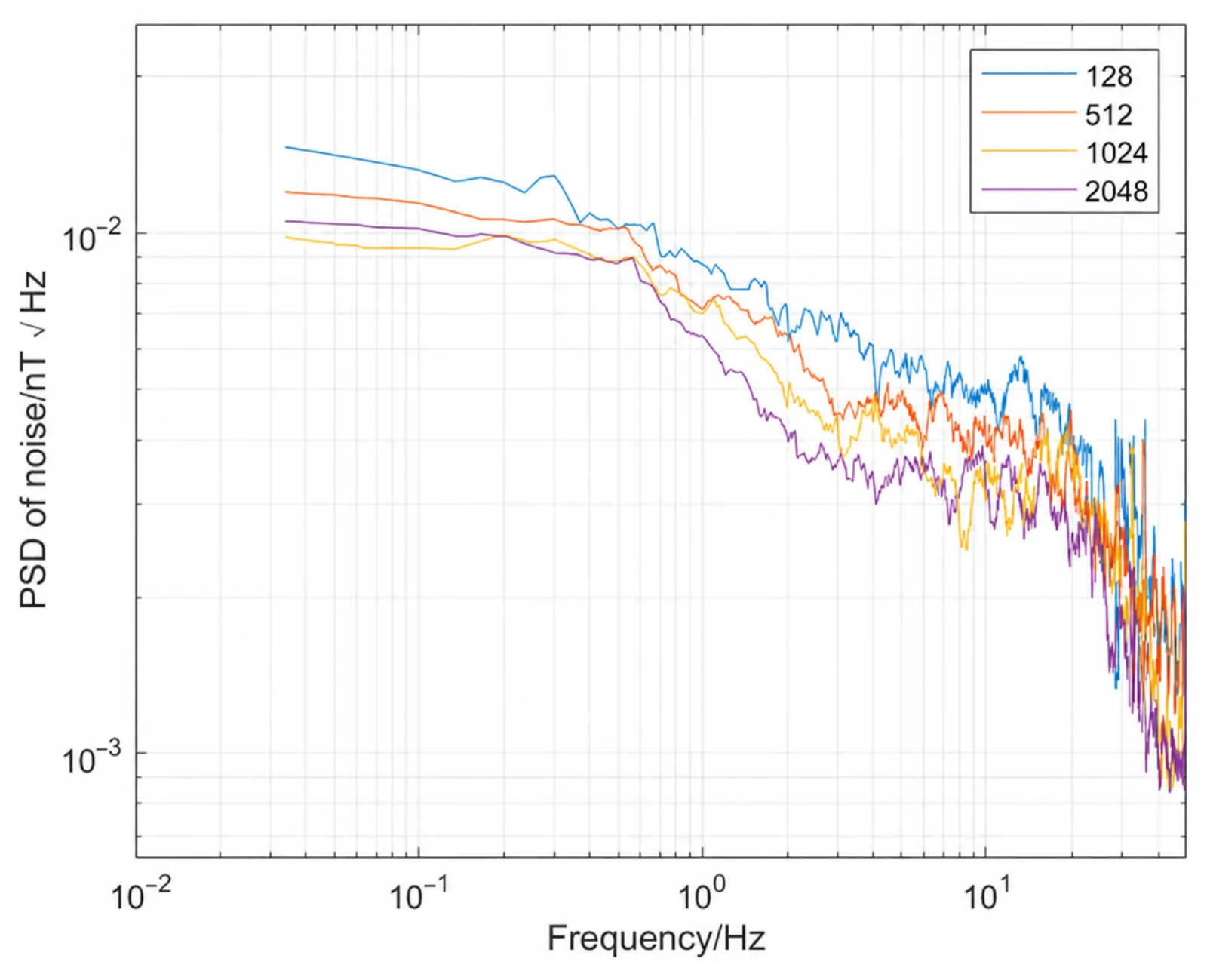
Noise power spectral density for four OSR configurations.

Figure 13 shows the noise power spectral density measured under an OSR = 2048 configuration. The noise power spectral density is primarily concentrated in the low-frequency range and gradually decreases with increasing frequency. The noise power spectral density at 1 Hz was 5.7 pT·Hz^−1/2^. To compare noise performance under different OSR configurations, noise measurements were performed for the four different OSR configurations. Compared to OSR = 128, the noise RMS at OSR = 2048 decreased from 0.151 to 0.106 nT. As shown in Figure 14, the noise power spectral density at 1 Hz decreased from 7.7 to 5.7 pT·Hz^−1/2^.

## 4. Discussion

Experimental results show that as the OSR increased from 128 to 2048, system non-linearity decreased from 0.050% to 0.024%. The noise RMS decreased from 0.151 nT to 0.106 nT, and the noise power spectral density at 1 Hz decreased from 7.7 to 5.7 pT·Hz^−1/2^. On the same magnetometer test platform, these results indicate that the OSR configuration affects both system non-linearity and low-frequency noise; a higher OSR can improve the overall measurement performance of the 1-bit Sigma–Delta digital feedback fluxgate magnetometer. This is consistent with the current trends in the development of fluxgate magnetometers for weak magnetic field detection, where noise reduction and improvements in measurement performance remain important research topics [2]. Therefore, adjusting the OSR not only can be used to optimize modulator design parameters but also serves as a system-level optimization method to enhance the system linearity and low-frequency noise performance.

**Figure 15:**
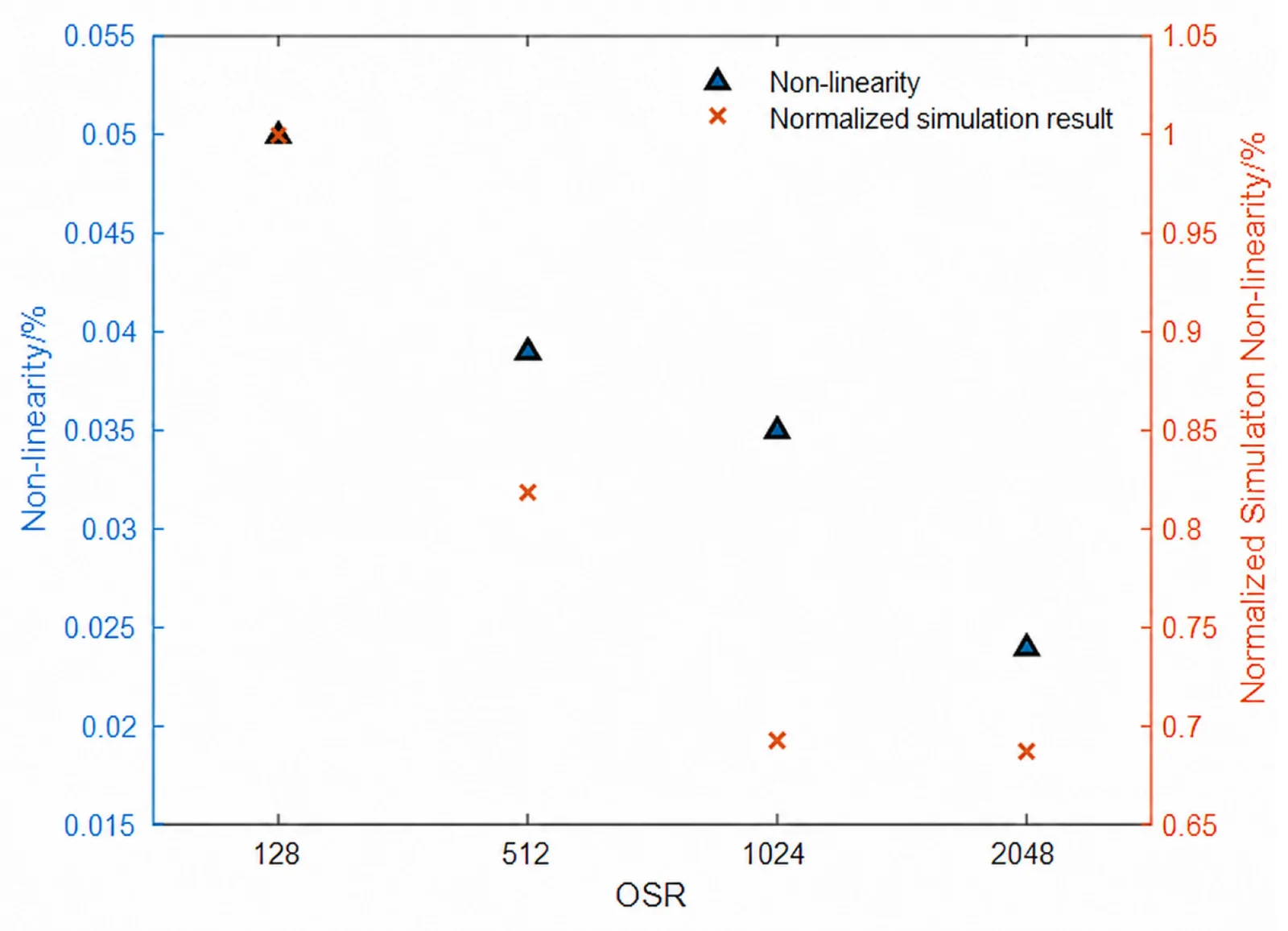
Non-linearity and normalized simulation results for four OSR configurations. The left Y-axis represents the measured non-linearity, marked as triangles; the right Y-axis represents the normalized simulation results, marked as crosses.

**Figure 16:**
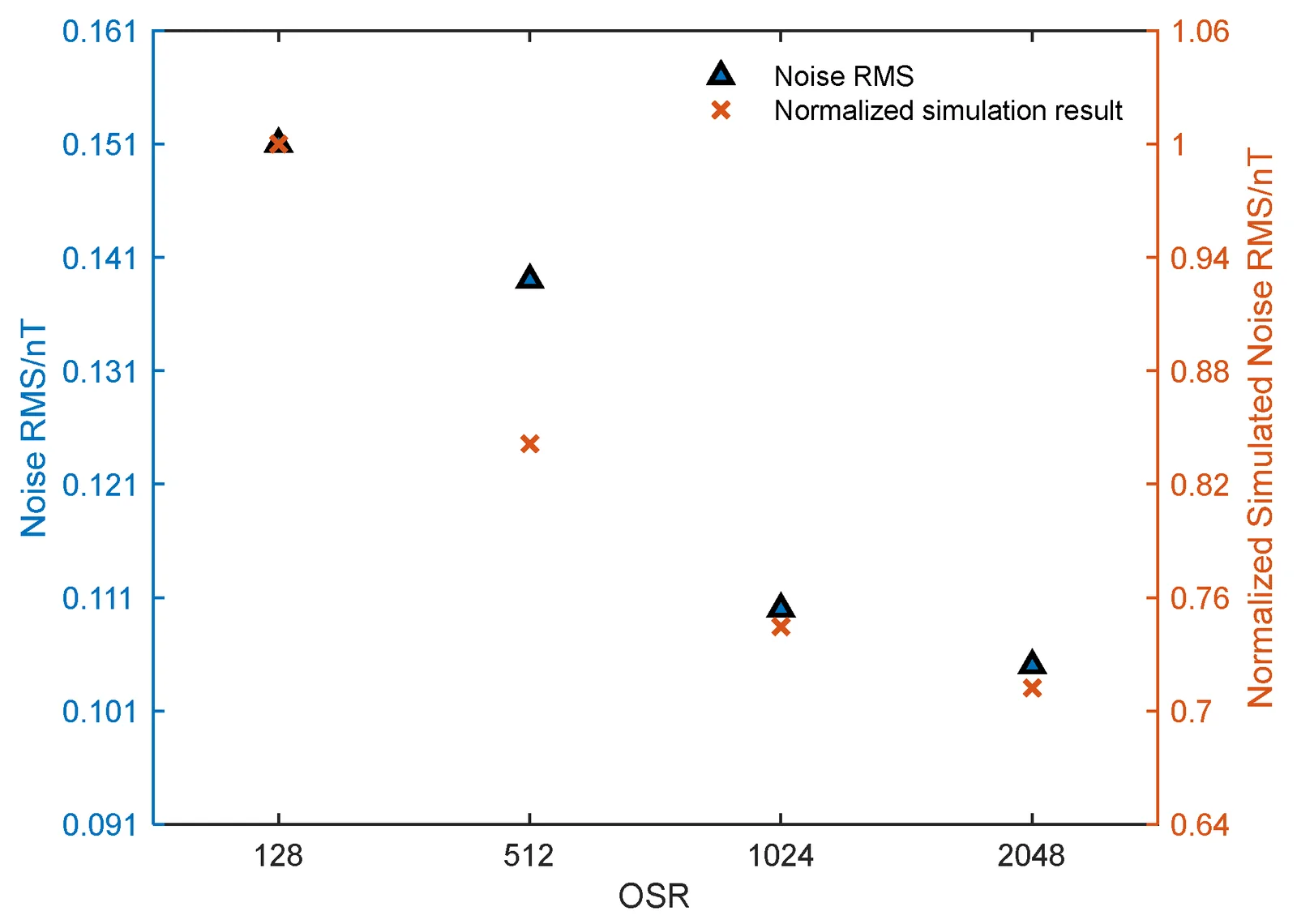
Noise RMS and normalized simulation results for four OSR configurations. The left Y-axis represents the measured non-linearity, marked as triangles; the right Y-axis represents the simulation results normalized to the test result at OSR = 128, marked as crosses.

Figure 15 and Figure 16 compare the simulation results with the experimental results for system non-linearity and noise RMS. The simulation model was developed based on the 1-bit Sigma–Delta digital feedback fluxgate magnetometer, using the same system architecture and main module parameter settings, with four different OSR configurations. For the linear simulation, constant magnetic fields were applied to the model at equal intervals across the full-scale range, and the system non-linearity for each OSR was calculated based on the model output. For the noise simulation, a constant magnetic field was applied to the model, and the noise RMS was calculated using the output fluctuations under this fixed input condition.

### 4.1. Non-Linearity Property

As shown in Figure 15, both the measured system non-linearity and the normalized simulation results decreased with higher OSR. When the OSR increased from 128 to 2048, the loop gain of the fifth-order single-loop 1-bit CRFB Sigma–Delta modulator increased from 5.81 × 10^5^ to 6.07 × 10^11^. Table 5 lists the loop gains calculated for four different OSR configurations.

This trend was consistent with the non-linear system test results. For a 1-bit digital feedback fluxgate magnetometer, increasing the OSR to achieve a higher loop gain meant that the feedback path could more effectively compensate for variations in the input magnetic field. After the feedback compensation capability was enhanced, the residual magnetic field error between the input magnetic field and the feedback magnetic field was reduced [23], thereby making the linear relationship between the system output and the input magnetic field more stable and further improving the system linearity.

### 4.2. Noise Property

As shown in Figure 16, both the noise RMS and the normalized simulation results decreased with higher OSR. The feedback quantization noise was pushed to the high-frequency band under higher-OSR conditions, thereby reducing the residual feedback quantization noise within the measurement frequency band. In higher-OSR conditions the low-frequency noise was dominated by the inherent noise of the fluxgate sensor and amplifier and residual noise from the test environment [24].

Table 6 compares the noise levels of the proposed system with several fluxgate magnetometers used for space missions. The noise level of the Solar Orbiter magnetometer was 10 pT·Hz^−1/2^ [25], while the analog fluxgate magnetometer of MMS in the ±500 nT low-range mode had a noise level of approximately 5 pT·Hz^−1/2^ at 1 Hz [26]. The system designed in this study had a noise power spectral density of 5.7 pT·Hz^−1/2^ within the measurement range of ±65,000 nT at 1 Hz. This result indicated that the system had the potential to achieve wide-range, low-noise magnetic field measurements, making it especially suitable for high-precision magnetic field measurements in Earth’s magnetic field.

In the application of space magnetic field measurement, high-sensitivity sensors are required to detect the minute changes in the magnetic field in the space environment [29]. Low-frequency noise limits the minimum detectable magnetic field change, and system non-linearity introduces measurement errors. Therefore, considering constraints such as modulator stability, system bandwidth, hardware resources, and power consumption, a digital magnetometer using a 1-bit Sigma–Delta modulator should have a higher OSR to reduce measurement uncertainty caused by low-frequency noise and non-linear errors.

## 5. Conclusions

This paper presents a digital feedback fluxgate magnetometer using a fifth-order single-loop 1-bit Sigma–Delta modulator. The test results show that increasing the OSR improves the system quantization noise within the signal bandwidth and reduces the residual error in the feedback signal, thereby enabling the system to achieve excellent linearity and low-frequency noise performance across a wide magnetic field range. In the proposed test system, optimal performance was achieved when OSR = 2048 with a non-linearity of 0.024% and a noise power spectral density of 5.7 pT·Hz^−1/2^ at 1 Hz within the ±65,000 nT measurement range. These results demonstrate that this digital fluxgate magnetometer can achieve high linearity and low noise levels in Earth magnetic field measurements.

The fifth-order single-loop 1-bit Sigma–Delta structure used in system combines the advantages of high-order noise shaping and 1-bit structure. In terms of structure, the fifth-order single-loop modulator has strong low-frequency quantization noise suppression; meanwhile, the 1-bit output helps reduce the static non-linearity caused by component mismatch, thereby further improving the system linearity and low-frequency noise performance.

Based on the above OSR performance improvement, further optimization of the OSR configuration while maintaining loop stability and system bandwidth can effectively enhance the overall performance of the digital feedback fluxgate magnetometer to satisfy the scientific requirements for high-precision Earth and deep space magnetic field measurements.

## Figures and Tables

**Table 1:** Comparison of performance characteristics of different types of magnetometers.

Magnetometer Type	Sensitivity (V/T)	Resolution (nT/√Hz)	Power (mW)	Bandwidth (kHz)
Hall	50	130	35	17 [4]
AMR	160	2.7	-	5000 [5]
GMR	725	-	100	75 [6]
TMR	1200	5	14.4	5000 [7]
GMI	4000	15	-	10 [8]
Fluxgate	80,000	0.005	40	0.1 [9]

**Table 2:** Coefficients of the fifth-order 1-bit Sigma–Delta modulator under different OSRs.

OSR	128	512	1024	2048
a1	0.000657	0.000656	0.000656	0.000655
a2	0.008837	0.008866	0.008867	0.008867
a3	0.055509	0.055544	0.055545	0.055546
a4	0.251643	0.252100	0.252123	0.252128
a5	0.555704	0.555565	0.555558	0.555556
b1	0.000657	0.000656	0.000656	0.000655
g1	0.000175	0.000011	0.000003	0.000001
g2	0.000495	0.000031	0.000008	0.000002

**Table 3:** Key parameters of the magnetometer system.

System Parameter	Value
Input magnetic field range (nT)	±65,000
Excitation signal frequency (kHz)	10
Sigma–Delta modulator order (stages)	5
Sigma–Delta modulator bit depth (bit)	1
Sigma–Delta modulator oversampling ratio	128/512/1024/2048
Sample ratio (Hz)	100

**Table 4:** System non-linearity for four OSR configurations.

Oversampling Ratio (OSR)	System Non-Linearity (%)
128	0.050
512	0.039
1024	0.035
2048	0.024

**Table 5:** Calculated loop gain for four different OSR configurations.

Oversampling Ratio (OSR)	Calculated Effective Feedback Gain
128	5.81 × 10^5^
512	5.94 × 10^8^
1024	1.90 × 10^10^
2048	6.07 × 10^11^

**Table 6:** Magnetometer noise levels at 1 Hz.

Exploration Mission	Range (nT)	Noise PSD at 1 Hz (pT·Hz^−1/2^)
This paper	±65,000	5.7
Solar Orbiter	±128	<10 [25]
MMS	±500	~5 [26]
Venus Express	±524	<10 [27]
Rosetta	±16,384	~22 [28]

## Data Availability

The original contributions presented in this study are included in the article. Further inquiries can be directed to the corresponding authors.
